# Formulation and Physical Stability of High Total Solids Lentil Protein-Stabilised Emulsions for Use in Plant Protein-Based Young Child Formulae

**DOI:** 10.3390/foods12091741

**Published:** 2023-04-22

**Authors:** Nicolas Malterre, Francesca Bot, James A. O’Mahony

**Affiliations:** 1 School of Food and Nutritional Sciences, University College Cork, T12 Y337 Cork, Ireland; 2 Department of Food and Drug, University of Parma, 43100 Parma, Italy

**Keywords:** emulsion, lentil protein, infant formula, high total solids, physical stability

## Abstract

The demand for high-quality plant protein products is increasing and the aim of this work was to evaluate the impact of increasing the total solids content on the formation and stability of lentil protein stabilised oil-in-water emulsions. A series of emulsions were formulated using different proportions of total solids: 23, 26, 29, 32, and 35% (*w*/*v*). The emulsions were formulated using three ingredients—lentil protein, sunflower oil, and maltodextrin—which made up 15.85, 27.43, and 56.72% (*w*/*w*) of the total solids, respectively. The changes in apparent viscosity, particle size distribution, and colour during thermal processing were evaluated, with the physical stability investigated using an analytical centrifuge. The apparent viscosity of the solutions increased with total solids content (25.6 to 130 mPa.s^−1^), as did redness colour intensity (a* value increased from 5.82 ± 0.12 to 7.70 ± 0.09). Thermal processing resulted in greater destabilisation for higher total solids samples, as evidenced by greater changes in particle size, along with decreased redness colour. These results bring a better understanding of high total solids plant protein emulsions and factors affecting their stability, which could be used for the development of cost-effective and sustainable processing solutions in the production of plant protein young child formulae.

## 1. Introduction

The European plant-based food market has experienced a significant sales growth of 49% between 2018 and 2020 [1]. Indeed, diets have evolved throughout the years with increasing awareness of the environmental impact of the food system [2] as well as allergies and intolerances associated with animal-based diets [3,4]. Collectively, these considerations have stimulated research activities into plant proteins. Multiple studies have been performed in recent years in response to these needs of consumers, such as Vogelsang-O’Dwyer et al. (2021) [5], who investigated the suitability of pulse proteins in the production of high nutritional value plant-based milk alternatives, or Alonso-Miravalles et al. (2022) [6], who investigated the suitability of lentil protein for the processing and preparation of an infant nutritional product. Concerning infant nutritional products specifically, the protein ingredients used must meet specific requirements such as a high protein concentration to meet the growth and maintenance requirements of the infant, as well as low fibre and antinutritional compound concentrations to not impair the digestibility and overall nutritional quality of the formulated product. To meet these requirements, a number of different plant protein sources have been studied in recent years, with the most widely commercially available being soy-based formulae (FAO/WHO) or rice-based formulae for specific medical requirements [7].

Although there is a lack of characterisation in the scientific literature and very limited commercial availability of alternative proteins, some protein sources, such as pulses, represent a good alternative to animal-based proteins for infant nutritional products as they contain a relatively high concentration of protein (20–30%) and amphiphilic protein with good ability to form thick interfacial layers around oil droplets, conferring good emulsification properties on such proteins [8,9,10,11]. More specifically, lentil proteins have shown good emulsifying properties as well as good heat and pH stability [12,13]. On the other hand, the presence of antinutritional factors (e.g., trypsin inhibitors, condensed tannins, and phytic acid) has made the use of lentil protein for infant formula manufacture challenging [14]. Therefore, recent studies have investigated sustainable membrane filtration and isoelectric precipitation approaches to produce lentil protein isolate with high protein content (>90%) and low levels of antinutritional compounds ([15]). Other studies have also recently focused on the formulation and functionalities of lentil protein matrices; Jeske et al. (2019) [16] studied the suitability of lentil protein to produce milk substitutes with high protein content (3.3% *w*/*w*), whereas Alonso-Miravalles et al. (2022) [6] studied the suitability of lentil–quinoa protein blends for the formulation of stable oil-in-water emulsions, showing that mixtures of plant proteins including lentils have a positive effect on the physical stability and nutritional profile of the resultant emulsions.

Although the formulation of lentil protein-stabilised emulsions is being studied and the interest is increasing, studies investigating technological and processing innovations to enable the use of lentil protein in the formulation of sustainable, nutrient dense, colloidally stable plant protein-based infant formulas are extremely limited. Indeed, research has been conducted to improve the processing of dairy infant formulae, including the work of Vanga et al. (2018) [17] and Pires et al. (2020) [18], which investigated new heating technology to improve the thermal stability of heat-labile ingredients. Other innovations have focused on reducing the energy consumption of infant formula manufacture processes, including the work of Ramirez et al. (2006) [19] that showed that almost 90% of the energy consumption is the result of the energy intensive water removal to obtain a powder. Additionally, it has been shown that an increase of 2% total solids in the feed concentrate prior to spray-drying could lead to a reduction of 6% of energy consumption [20]. Therefore, some researchers have investigated technologies to increase the total solid concentration of the feed and/or improve the evaporation step. Chamberland et al. (2020) [21] investigated the suitability of pre-concentration technologies to decrease the amount of drying necessary such as pre-concentration using membrane technology involving the separation of the water from the product. Others have investigated novel technology to recombine the ingredients at higher solids and reduce the necessity for water removal downstream, such as Murphy et al. (2011) [22], who developed a high-solids steam injection technology to increase the colloidal stability (reducing protein denaturation) of high-solids emulsions, allowing for spray-drying while by-passing the evaporation step, which significantly reduced the cost of infant formula production. These studies have shown that reducing the water removal pre-drying through different technologies considerably reduced energy consumption and improved the energy efficiency of the existing processing lines. However, these studies have been mainly performed on dairy and animal-based protein systems. To the authors knowledge, in the literature, no information is available on plant-based emulsions at high levels of total solids (25–35% total solids). Indeed, in the area of plant proteins and more particularly lentil protein, most of the research is focused on diluted systems [6,12,15,16] that do not allow prediction of the performance of the protein ingredients at high levels of total solids.

The aim of this study was to investigate the effect of increasing the total solids content (in the range 23 to 35%) on the thermal and physical stability of lentil-protein stabilised oil-in-water emulsions. This is necessary to predict the quality and stability of high total solids content emulsions during evaporation and spray-drying, underpinning more energy efficient conversion of concentrated emulsions into young child formula powders with tailored functional properties.

## 2. Materials and Methods

### 2.1. Materials

Red lentil protein isolates with a protein content of 79.63% (*w*/*w*) were acquired from the Fraunhofer Institute (Munich, Germany). Sunflower oil was obtained from a local commercial outlet (Tesco, Welwyn Garden City, Hertfordshire, UK), whereas maltodextrin with a dextrose equivalent value of 17 (Glucidex^®^ 17) was acquired from Roquette Frères (Lestrem, France). All reagents used in the study were of analytical grade and sourced from Sigma-Aldrich (St. Louis, MO, USA) unless explicitly specified.

### 2.2. Preparation of Oil-in-Water Emulsions

Model young child formula emulsions containing 15.85, 27.43, and 56.72% red lentil protein, sunflower oil, and maltodextrin, respectively, on a total solid basis, were prepared as follows. The red lentil protein was dispersed in pre-heated deionised water (70 °C) and stirred using magnetic stirring at 300 rpm for 1 h at 22 °C, followed by the addition of maltodextrin and stirring magnetically for an additional 2 h at 300 rpm and 22 °C. The solution was adjusted to pH 6.8 and allowed to rehydrate fully at 4 °C for 18 h, after which the temperature of the aqueous solution was adjusted to 22 °C and the pH was adjusted to 6.8 before adding sunflower oil. The mixture was heated to 50 °C in a water bath and an Ultra-Turrax (T25 Ultra-Turrax, Staufen, Germany) was used (12,000 rpm for 3 min) to obtain a coarse emulsion. The coarse emulsion was then passed through a two-stage valve homogeniser (APV 1000, SPX flow, Charlotte, NC, USA) in two passes at 180 bar total pressure, with first and second stage pressures of 150 and 30 bar, respectively. The homogenised emulsions were cooled to room temperature and analysed on the same day. In all formulations, the lentil protein, sunflower oil, and maltodextrin ratio was maintained constant, whereas five different total solids (TS) contents of 23, 26, 29, 32, and 35% TS were prepared as described above.

### 2.3. Colour of Lentil-Protein-Stabilised Emulsions

The colour of the emulsions before and after heating at 90 °C for 120 s (see Section 2.4) was assessed using a Chroma Meter CR-400 (Konica Minolta Sensing, Inc., Osaka, Japan) equipped with a liquid cell, using the CIELAB coordinates L*, a*, and b*, corresponding respectively to the brightness (ranging from 0, black, to 100, white), green to red (negative values being green, positive values being red), and blue to yellow (negative values being blue, positive values being yellow). Before the analysis, a calibrated white tile was used for calibration.

### 2.4. Thermal Stability and Heat-Induced Changes in Viscosity

An AR-G2 controlled-stress rheometer (TA Instruments Ltd., Waters LLC, Leatherhead, Surrey, UK) equipped with a starch pasting cell geometry was used to determine the changes in viscosity during the heat treatment of emulsions with increasing TS. The rheometer cell had an internal diameter of 36.0 mm, whereas the rotor diameter was 32.4 mm and the gap between the two elements at the base was 5.5 mm. The sample (28 g) was weighed into the cell before being successively conditioned and held at 30 °C for 120 and 300 s, respectively, after which the temperature was raised to 90 °C at a rate of 10 °C/min and held at 90 °C for 120 s. The temperature was then decreased to 30 °C at a rate of 10 °C/min and held at 30 °C for 300 s. Throughout the analysis, a fixed shear rate of 10 rad/s was maintained, and viscosity was measured continuously.

### 2.5. Particle Size Distribution

The particle size distribution (PSD) of the emulsions was measured before and after heat treatment at 90 °C for 120 s using a Mastersizer 3000 static light scattering instrument equipped with an automated Hydro MV wet cell disperser (Malvern Instruments, Worcestershire, UK). The refractive index of protein was set at 1.45, the absorption refractive index used was 0.001, and the dispersant refractive index was 1.33. The emulsions were measured at 20 °C and dispersed into ultrapure water until a laser obscuration of 10% was reached. Data were presented as volume-distribution-based particle size Dx(50) (particle size below which 50% of sample volume is found) and peak range (width of the particle size distribution for each peak).

### 2.6. Confocal Laser Scanning Microscopy

Microstructural analysis of the emulsions was performed using an OLYMPUS FV1000 confocal laser scanning biological microscope (Olympus Corporation, Tokyo, Japan). The emulsions before and after heat treatment at 90 °C for 120 s were prepared as described by Alonso-Miravalles et al. (2020) [23] with the following modifications: 1 mL of the emulsion was mixed in 4 mL of low-gelling-temperature agarose (Sigma-Aldrich, St. Louis, MO, USA) solution (1.5%, *w*/*v*) at 37 °C to fix the oil globules and allow for higher-quality imaging. As described by Grasso et al. (2021) [24], a mixture of the two stains, Fast Green FCF aqueous solution (200 μL of 0.1 g/L) and Nile Red in 1,2-propanediol (600 μL of 0.1 g/L), was prepared, and 50 μL of the mixture was added to 1 mL of the emulsion–agarose solution, which was then incubated at 20 °C until gelation. Fast Green FCF and Nile Red were excited at 633 and 488 nm, respectively [25], and representative images, performed using a 40× objective lens, were reported where fat and protein were stained green and red, respectively.

### 2.7. Accelerated Physical Stability Analysis

The stability of the emulsions was measured using an analytical centrifuge (LUMiSizer^®^, LUM GmbH, Berlin, Germany). The samples where centrifuged at 3 successive speeds: 117 g for 60 min, 1057 g for 60 min, and 1878 g for 16.7 min [26]. Near-infrared light illuminated the samples throughout the analysis and the intensity of the transmitted light over the entire length of the sample was measured every 10 s. The separation rate was determined by plotting the integral of the intensity of the transmitted light over time and extracting the slope (%/h). Additionally, the thickness (i.e., height) of the cream layer (mm) as well as the thickness (i.e., height) of the sediment layer (mm) were measured on the last profile for each sample.

### 2.8. Statistical Data Analysis

All samples were formulated in independent triplicate trials and all analyses were conducted in triplicate, unless otherwise stated. The data generated was subject to one-way analysis of variance (ANOVA) using SPSS (IBM SPSS statistics for Windows, version 28, IBMCorp, Armonk, NY, USA). To determine whether there were statistically significant differences (*p* < 0.05) between the mean values of different samples at a 95% confidence level, a Tukey’s paired comparison test was used.

## 3. Results

### 3.1. Colour of Lentil Protein-Stabilised Emulsions

The colour parameters of the lentil protein-stabilised emulsions at 23% TS were 78.37, 5.82, and 10.85 for L*, a*, and b*, respectively (Table 1). The brightness (L*) values significantly (*p* < 0.05) decreased, whereas the a* values, which are generally associated with red colour, significantly (*p* < 0.05) increased with increasing TS from 23 to 35%. This is expected for food systems formulated with high proportions of red lentil, which contains polyphenolic compounds such as tannins, phenolic acids, and anthocyanins [27]. When the lentil protein-stabilised emulsions were subjected to a thermal treatment of 90 °C for 120 s, the brightness values (L*) increased in all the samples, whereas a significant (*p* < 0.05) decrease in a* values was observed in all samples. This is likely due to degradation of polyphenolic compounds (i.e., tannins, phenolic acids, and anthocyanins) upon thermal treatment [28,29].

### 3.2. Apparent Viscosity of the Solutions during Thermal Processing

**Figure 1:**
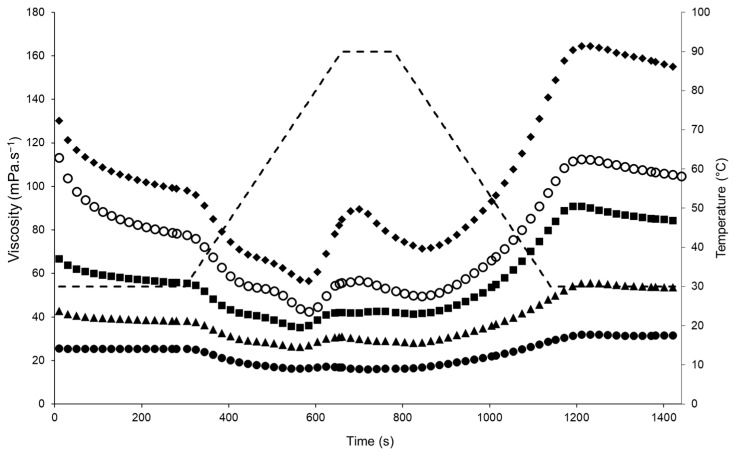
Apparent viscosity profiles of lentil protein-stabilised emulsions prepared at total solids contents of 23 (●), 26 (▲), 29 (■), 32 (○), and 35% (◆) during heat treatment with a peak hold at 90 °C for 120 s using a starch pasting cell geometry on a controlled-stress rheometer. Dashed line (- -) represents the temperature profile.

The initial apparent viscosity of the emulsions before heating (30 °C) increased with increasing TS, with the 23, 26, 29, 32, and 35% TS samples having mean apparent viscosity values of 25.6, 42.8, 66.6, 113, and 130 mPa.s^−1^, respectively (Figure 1). During heating from 30 to 75 °C, the viscosity decreased, as expected for emulsions [30] or protein suspensions; at 75 °C, which corresponds to the denaturation temperature of lentil proteins as reported by Barbana and Boye (2013) [31], a sharp increase in viscosity was observed. Similar viscosity profiles have been reported for oil-in-water emulsions stabilised by lentil protein in the range 25–30% TS [23] and in emulsions stabilised by hydrolysed whey proteins [30]. An increase in the viscosity of protein-stabilised emulsions at a relatively high temperature (70 °C) is attributed to the unfolding of polypeptide chains, disruption of hydrophobic interactions, and aggregation of unfolded protein molecules through covalent and non-covalent bonding [10,30,32,33]. The greatest heat-induced changes in viscosity were observed for the emulsion with 35% TS, with the viscosity increasing from 56.2 mPa.s^−1^ at 75 °C to 89.6 mPa.s^−1^ at 90 °C. During cooling, the viscosity reached final values of 31.5, 53.6, 84.0, 105, and 155 mPa.s^−1^ for the emulsions at 23, 26, 29, 32, and 35% TS, respectively. Similar viscosity values have also been observed in oil-in-water emulsions stabilised using lentil protein isolate in the range 25–30% TS [23]. Although the focus of the present study is on formation and stability of protein-stabilised emulsions, protein–protein interactions (e.g., unfolding and aggregation) between unadsorbed proteins in the continuous phase would also be expected to contribute strongly to the measured changes in viscosity during heating [34].

### 3.3. Particle Size Distribution

**Figure 2:**
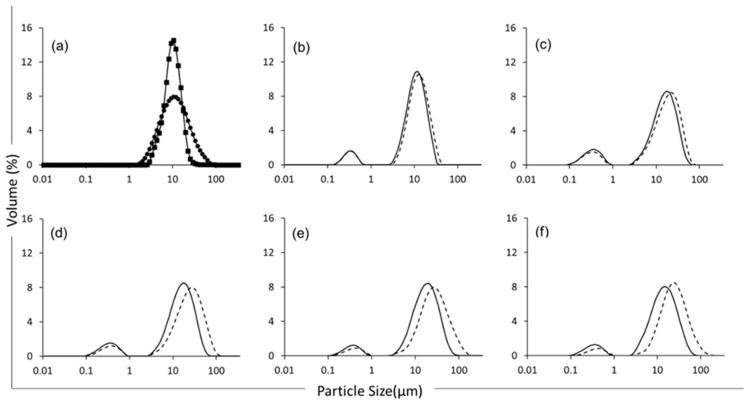
Particle size distribution of lentil protein isolate powder (-●-), lentil protein isolate dispersion (-■-) (**a**) and lentil protein-stabilised emulsions at total solids contents of 23% (**b**), 26% (**c**), 29% (**d**), 32% (**e**) and 35% (**f**), before (solid line) and after (dashed line) heat treatment at 90 °C for 120 s.

Bimodal particle size distributions were observed in all emulsion samples (Figure 2). The population of smaller particles (Peak 1, corresponding to particles in the range 0.1–1 µm) can be associated with discreet oil droplets formed through homogenisation and stabilised by the lentil proteins, with Dx(50) (particle size below which 50% of sample volume is found) values of 0.34, 0.36, 0.36, 0.38, and 0.35 µm for the emulsions at 23, 26, 29, 32, and 35% TS, respectively. The results are in accordance with those of Alonso-Miravalles et al. (2020) [23], while formulating lentil protein-stabilised emulsions with similar protein-to-oil ratios (1:1.71). The population of larger particles (Peak 2, corresponding to particles in the range 2 to 80 µm) had Dx(50) values of 12.3, 17.9, 18.6, 20.9, and 17.0 µm, for emulsions with 23, 26, 29, 32, and 35% TS, respectively. This second population is associated with insoluble plant material from the lentil protein ingredient that was not fully solubilised during the powder rehydration process and homogenisation used in this study (Figure 2a); indeed, particles in the size range 2–80 µm were present in both the lentil protein isolate powder and lentil protein isolate suspensions. In addition, the emulsion sample at 23% TS displayed a lower particle size range (Table 2), with a peak 1 range of 0.69 µm and a peak 2 range of 40.1 µm before heating, whereas the other samples displayed peak 1 ranges of 0.98, 0.99, 0.94, and 0.90 µm and peak 2 ranges of 64.3, 84.0, 117, and 92.6 µm for the samples with 26, 29, 32, and 35%TS, respectively. Overall, the samples showed a wider range of particle sizes at higher TS contents, which is indicative of lower-quality emulsions due to more heterogeneous oil globule size distributions.

The particle size distribution of the emulsions was also measured after heat treatment at 90 °C for 120 s (Figure 2). The results showed an increase in particle size for both populations of particles, with the particles having Dx(50) values of 0.34, 0.34, 0.38, 0.43, and 0.42 µm for Peak 1 and 14.1, 21.3, 28.8, 34.2, and 29.9 µm for Peak 2 for the emulsions with 23, 26, 29, 32, and 35% TS, respectively, indicating that heating caused some emulsion destabilisation. Although the size of the emulsified particles (i.e., Peak 1) increased for all samples, a greater increase in particle size was recorded for the higher TS samples (Table 2), as evidenced by the wider range in particle size. For example, the 35% TS emulsion displayed a Peak 1 range increase of 15.3%, whereas the 23% TS emulsion displayed a Peak 1 range increase of just 1.13%. 

Similarly, the second population of larger particles (i.e., Peak 2) underwent an increase in particle size that was more extensive for the higher TS emulsions, with range increases of 181% for the 35% TS emulsion and 28% for the 23% TS emulsion. In this regard, Euston et al. (2000) [35] found that an increase in TS in whey protein-based oil-in-water emulsions at protein contents ranging between 0.2 and 2% could lead to a decrease in the inter-droplet space, leading to increased flocculation upon heat treatment. These results are in agreement with those of Alonso-Miravalles et al. (2022) [6], who found similar results for lentil–quinoa protein blends thermally treated at 95 °C for 30 s.

### 3.4. Microstructure and Fat Distribution

**Figure 3:**
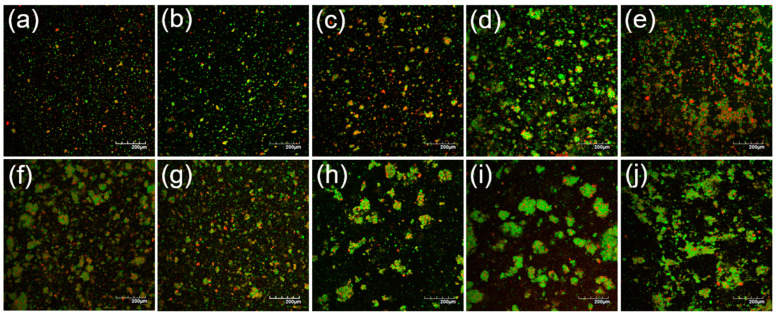
Confocal laser micrographs of lentil protein-stabilised emulsions before (**a**–**e**) and after (**f**–**j**) heat treatment at 90 °C for 120 s for the solutions at 23 (**a**,**f**), 26 (**b**,**g**), 29 (**c**,**h**), 32 (**d**,**i**), and 35% (**e**,**j**) total solid content; fat and protein were stained green and red, respectively.

Using confocal laser scanning microscopy analysis, the emulsions at 23% TS before heat treatment displayed small, uniformly distributed oil droplets (Figure 3). With increasing the TS content up to 35%, the concentration of emulsified oil droplets increased, as shown by the higher density of green particles on the micrographs. This is in agreement with the observations of Jeske et al. (2019) [16], who investigated the microstructure of lentil protein-stabilised emulsions homogenised at a similar pressure to that used in this study (i.e., 180 bar). In addition to green-labelled oil droplets, red-labelled protein-dense particles were also evident on the confocal micrographs in Figure 3; these increased in relative proportion with increasing the TS content from 23 to 35%. It is likely that these were mostly undissolved lentil protein powder particles. After heat treatment at 90 °C for 120 s, larger clusters of flocculated oil droplets were observed, in addition to protein-dense aggregates; this was in agreement with the particle size distribution (Figure 2) and viscosity (Figure 1) results. The greatest abundance of flocculated oil droplets and aggregated protein particles were evident in the high TS content samples (29, 32, and 35% TS). These results are in agreement with Primozic et al. (2017) [36], who investigated the stability of lentil protein isolate-stabilised oil-in-water nanoemulsions (0.1–5%, *w*/*w*, protein) and reported that higher concentrations of protein in the continuous phase led to stronger interactions between proteins at the interface and in the continuous phase, which caused the formation of protein networks and greater flocculation of oil droplets. Similarly, Tang and Ghosh (2021) [37], who worked with oil-in-water emulsions stabilised with canola protein isolate (1–4%, *w*/*w*, protein), found evidence that unadsorbed protein in the continuous phase allowed for the formation of protein networks that promoted aggregation of protein-coated droplets.

### 3.5. Accelerated Physical Stability

**Figure 4:**
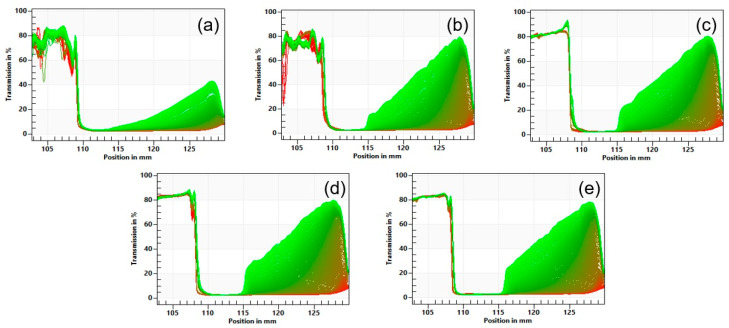
Representative accelerated stability analysis transmission profiles for the lentil protein-stabilised emulsions at 23 (**a**), 26 (**b**), 29 (**c**), 32 (**d**), and 35% (**e**) total solids content.

**Figure 5:**
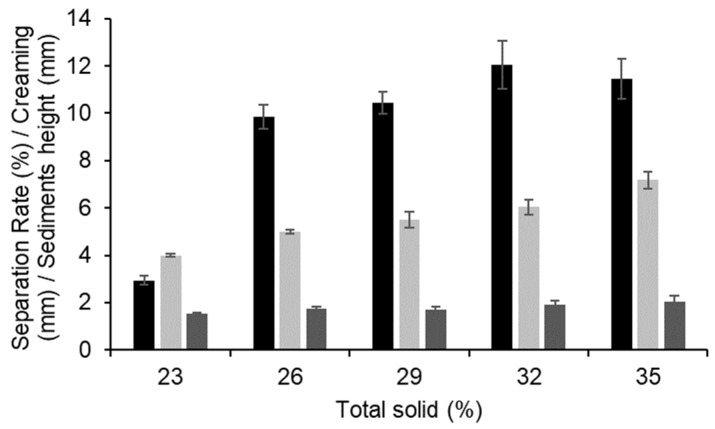
Separation rate (black), cream layer height (light grey), and sediment layer height (dark grey) of lentil-protein-stabilised emulsions at total solid contents of 23, 26, 29, 32, and 35% from accelerated stability analysis.

Representative transmission profiles during centrifugation for lentil protein-stabilised emulsions with TS in the range 23–35% are shown in Figure 4. The initial transmission profiles of the emulsions were similar among all samples, with integral transmission values ranging from 3.2 to 3.4%. All the emulsions showed a rapid increase in transmission on centrifugation, which was greater for emulsions with higher TS contents. Specifically, the separation rates were 2.93 ± 0.19, 9.84 ± 0.51, 10.46 ± 0.46, 12.03 ± 1.01, and 11.46 ± 0.85% for the emulsions with 23, 26, 29, 32, and 35% TS (Figure 5), respectively. Jeske et al. (2019) [16] found similar profiles and separation rates for emulsions stabilised with lentil protein formulated at 3.3% protein (*w*/*w*) and homogenised at 180 bar. No significant (*p* > 0.05) differences were observed in the sediment thickness between the samples, with values ranging between 1.52 and 2.02 mm. Furthermore, it was possible to observe differences among the samples in the thickness of the cream layer, which is the result of the upward motion of the oil phase. The thickness of the cream layer of the emulsions was 3.99, 4.99, 5.49, 6.02, and 7.17 mm for the emulsions with 23, 26, 29, 32, and 35% TS, respectively, indicating that with increasing TS content the emulsions were significantly (*p* < 0.05) less physically stable.

## 4. Conclusions

The physicochemical properties and heat stability of high total solids lentil-protein-stabilised oil-in-water emulsions were studied. The emulsions, ranging from 23 to 35% TS, showed bimodal particle size distributions in addition to increasing viscosity with increasing TS content. On heating at 90 °C for 120 s, the emulsions at low TS concentrations (i.e., 23–29%) showed better physical stability than emulsions at high TS concentrations. Microstructural analysis confirmed that these physical changes can be attributed to strong protein–protein interactions, which resulted in protein aggregation and ultimately oil droplet flocculation. These results bring a better understanding of high-TS plant protein emulsion stability and could be used for the development of cost-effective and sustainable processing solutions for the production of plant-protein young child formulae.

## Figures and Tables

**Table 1:** Colour space values of lentil protein-stabilised emulsions before and after heat treatment at 90 °C for 120 s.

TS (%)	Before Heat Treatment			After Heat Treatment		
	L*	a*	b*	L*	a*	b*
23	78.37 ± 0.56	5.82 ± 0.12	10.85 ± 0.25	80.00 ± 0.18	1.69 ± 0.03	9.60 ± 0.11
26	78.22 ± 0.52	6.30 ± 0.27	11.55 ± 0.01	79.46 ± 0.21	2.04 ± 0.07	10.25 ± 0.08
29	77.29 ± 0.17	6.93 ± 0.02	12.57 ± 0.04	78.41 ± 0.31	2.11 ± 0.02	11.21 ± 0.24
32	76.44 ± 0.36	7.20 ± 0.03	12.93 ± 0.14	77.36 ± 0.18	2.40 ± 0.10	11.64 ± 0.02
35	75.78 ± 0.13	7.70 ± 0.09	13.67 ± 0.13	76.42 ± 0.13	2.59 ± 0.08	12.47 ± 0.06

**Table 2:** Particle size distribution parameters of lentil protein-stabilised emulsions before (BH) and after heat treatment (AH) at 90 °C for 120 s.

		TS (%)				
		23	26	29	32	35
Dx(50) (μm)	Peak 1 BH	0.34 ± 0.01	0.36 ± 0.01	0.36 ± 0.02	0.38 ± 0.02	0.35 ± 0.02
	Peak 2 BH	12.3 ± 0.11	17.9 ± 3.05	18.6 ± 1.65	20.9 ± 3.27	17.0 ± 3.22
	Peak 1 AH	0.34 ± 0.01	0.34 ± 0.01	0.38 ± 0.03	0.43 ± 0.02	0.42 ± 0.01
	Peak 2 AH	14.1 ± 0.5	21.3 ± 2.98	28.8 ± 1.98	34.2 ± 3.14	29.9 ± 2.97
Range (µm)	Peak 1 BH	0.69 ± 0.03	0.98 ± 0.01	0.99 ± 0.05	0.94 ± 0.08	0.90 ± 0.07
	Peak 2 BH	40.1 ± 3.32	64.3 ± 11.5	84.0 ± 9.81	117 ± 24.21	92.6 ± 40.3
	Peak 1 AH	0.70 ± 0.01	0.95 ± 0.05	1.03 ± 0.07	1.04 ± 0.07	1.04 ± 0.08
	Peak 2 AH	51.4 ± 2.93	80.9 ± 11.5	158 ± 28.2	251 ± 57.3	232 ± 42.3
% Change in range on heat treatment	Peak 1	1.13	2.60	3.53	10.5	15.3
	Peak 2	28.1	25.9	89.0	112	181

Dx(50) = particle size below which 50% of sample volume is found. Range = width of the particle distribution.

## Data Availability

Data will be made available on request.
